# Non-steroidal Anti-inflammatory Drug Misuse: Knowledge, Attitudes and Practices of Community Pharmacists in Morocco

**DOI:** 10.7759/cureus.97559

**Published:** 2025-11-23

**Authors:** Nada Benzine, Hanan Rkain, Fatine Kronbi, Hind L'heri, Houda Sefiani, Ghita Benabdallah, Redouane Abouqal, Najia Hajjaj-Hassouni, Latifa Tahiri, Fadoua Allali

**Affiliations:** 1 Department of Rheumatology B, Ayachi Hospital, Ibn Sina Hospital Center, Rabat, MAR; 2 Faculty of Medicine and Pharmacy, Mohammed V University, Rabat, MAR; 3 Laboratory of Physiology, Faculty of Medicine and Pharmacy, Mohammed V University, Rabat, MAR; 4 Department of Pharmacology, Anti Poison and Pharmacovigilance Centre of Morocco, Rabat, MAR; 5 Department of Pharmacy, Anti Poison and Pharmacovigilance Centre of Morocco, Rabat, MAR; 6 Laboratory of Biostatistics, Clinical and Epidemiological Research, Faculty of Medicine and Pharmacy, Mohammed V University, Rabat, MAR; 7 Research Center of Health Sciences (CreSS), College of Health Sciences, International University of Rabat (UIR), Rabat, MAR

**Keywords:** community pharmacy services, drug misuse, morocco, non-steroidal anti-inflammatory agents, pharmacists

## Abstract

Objective

This study aimed to assess the level of knowledge, attitudes, and practices of community pharmacists in Morocco regarding the misuse of non-steroidal anti-inflammatory drugs (NSAIDs) and to identify factors influencing their management of such cases in daily practice.

Methods

A descriptive cross-sectional study was conducted among community pharmacists in Morocco using a structured, self-administered questionnaire distributed via Google Forms between February 10 and April 30, 2024. The questionnaire collected information on pharmacists’ sociodemographic characteristics, knowledge, attitudes, and practices regarding the misuse of NSAIDs. Knowledge and attitudes were assessed using a five-point Likert scale. The questionnaire was pre-tested for clarity and internal consistency. Data were analyzed using descriptive statistics and inferential tests (e.g., Chi-square) where applicable. Ethical approval was obtained from the Ethics Committee of Mohammed V University in Rabat, and participation was voluntary with informed consent.

Results

A total of 100 pharmacists participated in the survey. The mean age was 44 years, 55.3% were women, and the mean professional experience was 17 years. Approximately 60% demonstrated a clear understanding of NSAID misuse. Most were aware that NSAIDs can cause gastric ulcers (96%) and should be used with caution in individuals with cardiovascular conditions (83%). The main reasons cited for misuse were persistent or severe pain (82%) and lack of awareness of side effects (79%). Financial constraints were reported by 55% as a barrier to preventing misuse. In daily practice, 84% of pharmacists always informed patients of the maximum safe dose, 85% explained the recommended treatment duration, and 70% checked for health conditions that could make NSAID use unsafe before dispensing.

Conclusion

This study provides important insights into NSAID misuse in community pharmacies in Morocco and the challenges faced in addressing it. The findings indicate that patient education is a crucial factor in limiting misuse, and a multidisciplinary approach involving different healthcare professionals is necessary to effectively tackle this public health concern. Comparable studies in North African and Middle Eastern countries have reported similar challenges, highlighting insufficient patient education and limited pharmacist intervention as key contributors to NSAID misuse.

## Introduction

Non-steroidal anti-inflammatory drugs (NSAIDs) are widely used worldwide owing to their analgesic, antipyretic, and anti-inflammatory properties [1]. They are often prescribed for rheumatologic and musculoskeletal disorders, as well as in cases involving injuries, dental issues, and other medical conditions. As NSAIDs are available over the counter, their frequent use increases the risk of misuse.

Drug misuse is defined as the use of a medicine in a way that does not comply with its Summary of Product Characteristics (SmPC) [2]. According to the French Public Health Code, misuse refers to the intentional and inappropriate use of a medicine in a manner inconsistent with its marketing authorization (MA) or established good practice guidelines (Article R.5121-152 of the CSP) [3,4]. For NSAIDs, misuse may involve inappropriate indications, excessive dosages, prolonged duration, or unsuitable routes of administration.

In Morocco, NSAID misuse is common. However, inappropriate use of these medicines can result in serious adverse effects, particularly gastrointestinal complications (such as peptic ulcers, gastrointestinal perforation, or bleeding), renal damage (such as interstitial nephritis or acute kidney injury) [5,6], and cardiovascular complications, including hypertension, heart failure, myocardial infarction, or stroke. These risks are increased with prolonged use or high doses, and are further heightened in patients with cardiovascular disease or risk factors such as hypertension, diabetes, or obesity [7-9].

To mitigate these risks, health authorities, including the French National Agency for Medicines and Health Products Safety (ANSM), recommend prescribing NSAIDs at the lowest effective dose and for the shortest possible duration [10]. Despite these recommendations, inappropriate use continues to pose a significant public health challenge, highlighting the need to evaluate pharmacists’ knowledge and practices regarding NSAID misuse.

Community pharmacists play a crucial role in preventing NSAID misuse, as they are often the first healthcare professionals to dispense these over-the-counter medicines. They are responsible for informing patients about the associated risks and promoting safe use [11].

To date, little research has investigated community pharmacists’ knowledge, attitudes, and practices regarding NSAID misuse. In Morocco, the absence of specific guidelines for pharmacists to ensure the safe use of NSAIDs represents a significant gap.

The objective of this study was to assess community pharmacists’ knowledge, attitudes, and practices regarding NSAID misuse in Morocco and to identify gaps where targeted interventions could strengthen safe dispensing and patient counseling.

## Materials and methods

Study design and population

This was a descriptive observational study carried out by the Rheumatology Department B, in partnership with the Moroccan Association for Research and Support for Rheumatic Patients (AMRAR) and the Moroccan Poison and Pharmacovigilance Center (CAPM). The study focused on community pharmacists working in Morocco between February 10 and April 30, 2024.

Eligibility criteria

All community pharmacists working in Morocco, whether in the public or private sector, were included. There were no exclusion criteria.

Sampling method

This was an exploratory study that used a convenience sampling approach. We aimed to collect the maximum number of responses possible. Participation was voluntary, and some pharmacists declined to take part or were geographically distant. The questionnaire was distributed both in person and electronically (via email and WhatsApp) to a large number of community pharmacists whose contact information was obtained through the Pharmacists’ Association.

Data collection

Data were collected using a structured questionnaire distributed via a mixed approach. Some participants were interviewed directly by three rheumatologist investigators using a multiple-choice questionnaire (MCQ), while others, who were geographically distant or unavailable at their pharmacies, received the questionnaire electronically via Google Forms to self-complete. All responses received between February 10 and April 30 were included in the study, and every question had to be answered. The investigators recorded responses from interviews directly into the online survey system.

Questionnaire

The survey questionnaire was developed following an extensive review of previous studies, including systematic reviews and related surveys on the misuse of NSAIDs. It was designed by a multidisciplinary panel of five experts, consisting of three rheumatologists (two professors and one PhD-level specialist) and two professors of pharmacovigilance working in academic hospitals.

To ensure content validity, each expert independently evaluated the questionnaire for clarity, relevance, and alignment with the study objectives. The assessments were then discussed collectively, and revisions were made based on consensus. This process helped confirm that all items were both comprehensive and contextually appropriate.

A pilot test was conducted with ten community pharmacists who were not part of the final study sample. The purpose was to evaluate the clarity, comprehensibility, and feasibility of the questionnaire. Feedback from the pilot phase led to minor adjustments in the wording to enhance precision and readability. The average time required to complete the questionnaire during the pilot was approximately 10 minutes, confirming its practicality.

In the final version, all questions were mandatory to avoid missing data. Responses were carefully screened for internal consistency, and incomplete or contradictory answers were excluded from analysis. Although the forced-response design minimized missing data, the possibility of response bias was acknowledged as a limitation of the study.

The Final Questionnaire Had Four Parts

Sociodemographic section: Collected information about gender, age, workplace, and years of professional experience.

Knowledge section: Assessed pharmacists’ understanding of the definition of misuse, how NSAIDs are classified (Lists I, II, III), and their main contraindications.

Attitudes section: Explored how pharmacists view their role in preventing NSAID misuse, why patients misuse these drugs, the challenges pharmacists face, and what steps could reduce misuse.

Practices section: Looked at how often pharmacists dispensed NSAIDs without a prescription, whether they educated patients on the minimum effective dose, maximum treatment duration, and possible side effects. It also checked if they verified drug interactions and contraindications, what they did in cases of misuse, and whether they reported adverse effects to the CAPM.

To define adequate knowledge, positive attitude, and good practice, responses were scored systematically: each correct or recommended behavior was assigned one point, and the total scores were summed for each participant. Thresholds for categorization were established based on the median or predefined cut-offs: participants scoring above the threshold were classified as having adequate knowledge, a positive attitude, or good practice, while those below were classified otherwise. This method ensured transparency and consistency in the evaluation of pharmacist knowledge, attitudes, and practices.

Pharmacists’ knowledge was assessed using a five-point Likert scale, with each item scored as follows: 1, true; 2, probably true; 3, probably false; 4, false; and five, I don’t know. This scale was chosen to allow nuanced responses while still clearly distinguishing correct from incorrect knowledge. The items were carefully reviewed by the expert panel to ensure clarity and minimize potential confusion.

Participants were considered to have demonstrated sufficient knowledge if they correctly answered at least 70% of the items in the knowledge section of the questionnaire. Attitudes and perceived difficulties were also rated with a five-point Likert scale (1, strongly agree; 2, agree; 3, neutral; 4, disagree; 5, strongly disagree). Practices were measured using the five-point Likert scale (1, always; 2, often; 3, sometimes; 4, rarely; 5, never).

The custom-designed questionnaire was reviewed and approved by experts. It demonstrated good reliability and validity, with Cronbach’s alpha scores of 0.76 for the knowledge section, 0.75 for the attitude section, and 0.86 for the practice section (p < 0.001). The use of a five-point Likert scale for all sections was chosen to balance sensitivity and simplicity, allowing respondents to express varying degrees of agreement or certainty without excessive complexity. Alternative scales, such as four- or seven-point Likert scales, were considered, but the five-point format was selected as it is widely used in similar surveys, facilitates comprehension among participants, and avoids central tendency bias.

Statistical analysis

A descriptive analysis was done on the validated data. Qualitative data are shown as numbers and percentages, while quantitative data are shown as averages ± standard deviation (SD) or as medians with interquartile ranges.

For further analysis, the Chi-square test was used to compare practice-related variables among pharmacists, such as actions taken in cases of NSAID misuse, reporting of adverse effects, patient counseling, and verification of drug interactions and contraindications. A result was considered statistically significant if the p-value was less than 0.05.

All 100 responses were complete; there were no missing data. Each practice variable was carefully checked for completeness before analysis to ensure accuracy and reliability. Pharmacists’ practices were grouped into two categories: “Frequently adopted” included answers of “Always” and “Often.” “Rarely or never adopted” included answers of “Sometimes,” “Rarely,” and “Never.” All data were analyzed using IBM SPSS Statistics for Windows, Version 20 (Released 2011; IBM Corp., Armonk, New York, United States).

Ethical considerations

Participation in this study was entirely voluntary. All community pharmacists were informed about the study’s objectives and procedures through an information letter, and written informed consent was obtained from each participant before completing the questionnaire. The questionnaire was distributed both in person and electronically (via email and WhatsApp) to community pharmacists whose contact information was obtained through the Pharmacists’ Association. All responses were treated confidentially and anonymized in accordance with ethical guidelines. Participants were explicitly informed that they could withdraw from the study at any time without any consequences. Ethical approval for the study was obtained from the Ethics Committee of Mohammed V University in Rabat (Faculty of Medicine and Pharmacy, ethics approval reference: CERB 55-24), and the study adhered to the ethical principles of the 1964 Declaration of Helsinki and its later updates.

## Results

Sociodemographic characteristics

A total of 100 community pharmacists in Morocco answered the questionnaire. Their average age was 44 ± 12 years, and 54% were women. Participants had an average of 17 ± 11 years of professional experience in community pharmacies. Each participant represented a unique pharmacy; no two respondents worked at the same location, ensuring independent responses. Most pharmacists (83%) worked in urban areas, 8% in rural areas, and 9% in peri-urban areas. The detailed sociodemographic data are shown in Table 1.

**Table 1:** Sociodemographic characteristics of community pharmacists in Morocco *Average ± standard deviation; **n (percentage %)

Variable		N = 100
Age *		44 ± 12
Sex **	Female	54 (54)
Average years of professional experience *		17±11
Workplace location **	Urban area	83 (83)
	Peri-urban area	9 (9)
	Rural area	8 (8)
Socioeconomic level of the neighborhood n **	High	61 (61)
	Medium	15 (15)
	Low	24 (24)

Knowledge

**Figure 1:**
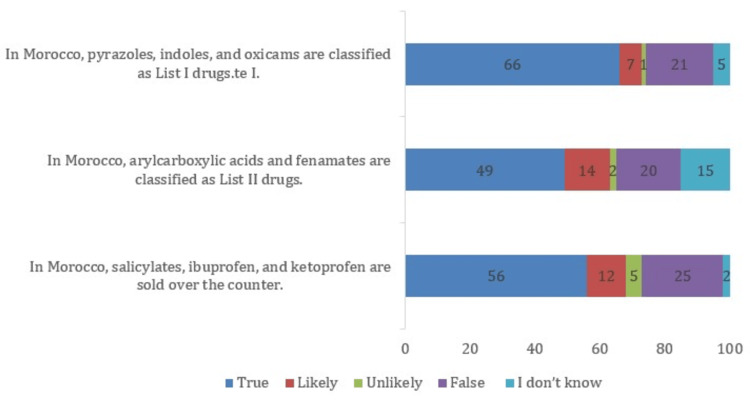
Knowledge of NSAID classification among community pharmacists in Morocco (N = 100) NSAIDs: non-steroidal anti-inflammatory drugs List I drugs (prescription-only NSAIDs): pyrazoles, indoles, and oxicams; List II drugs (pharmacy-controlled NSAIDs): arylcarboxylic acids and fenamates; Over-the-counter (OTC) NSAIDs: salicylates, ibuprofen, and ketoprofen

Sixty percent of pharmacists knew that misuse means the intentional and inappropriate use of a medicine that does not follow its MA. They understood that misuse includes using medicines for the wrong reason (75%), wrong dose (69%), or longer than recommended (62%). Only 32% could clearly tell the difference between misuse and accidental or unintentional wrong use. Pharmacists’ knowledge about the different regulatory lists of NSAIDs (List I, List II, and over-the-counter (OTC)) is shown in Figure 1.

Most pharmacists knew NSAIDs should be used carefully in patients at risk of gastroduodenal ulcers (96%) and those with cardiovascular risk (83%).

Attitudes

**Figure 2:**
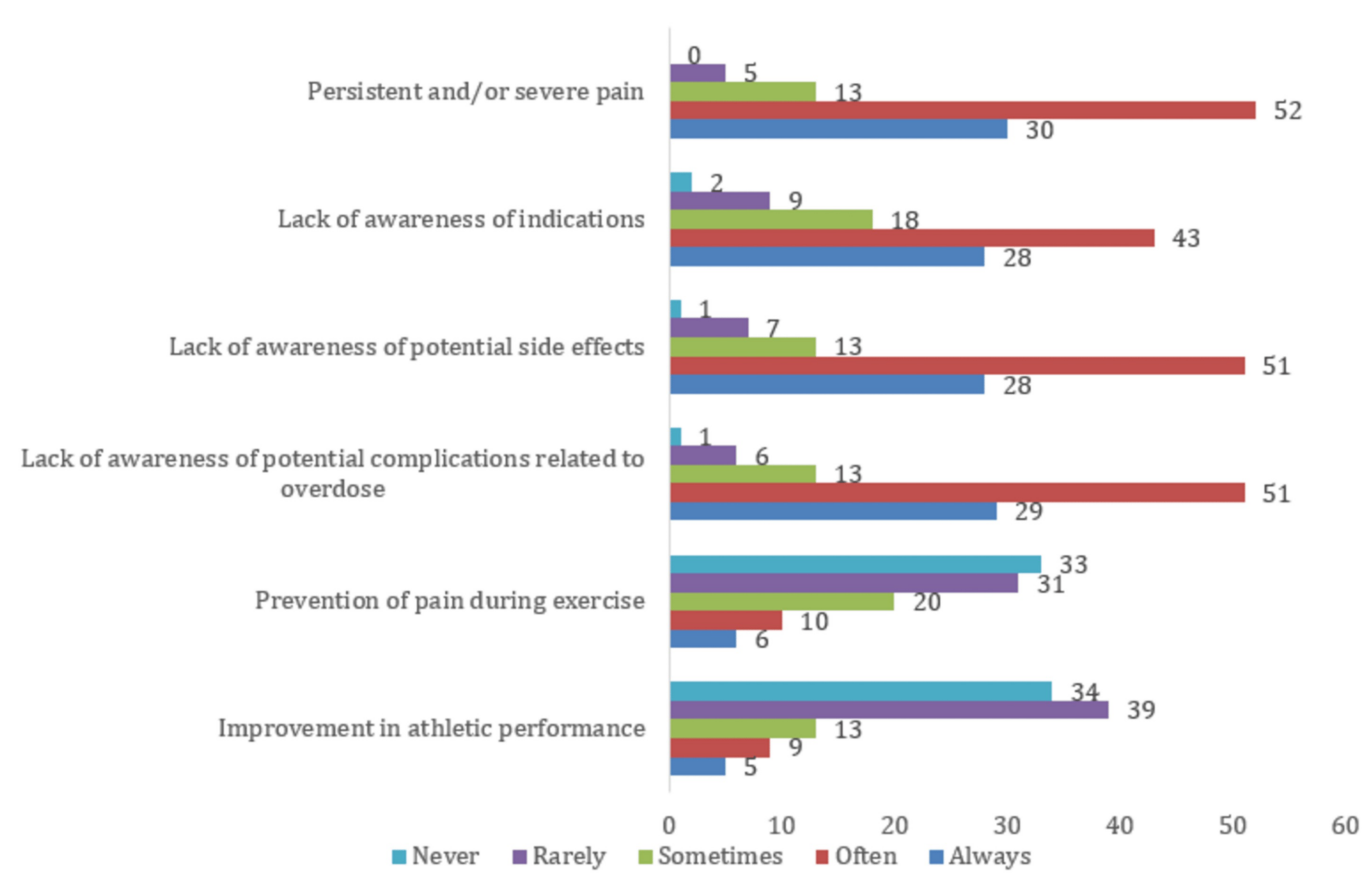
Reasons for NSAID misuse reported by patients, according to the community pharmacists in Morocco (N = 100) NSAID: non-steroidal anti-inflammatory drug

All pharmacists agreed that they have an important role in preventing NSAID misuse. Pharmacists’ views on why patients misuse NSAIDs are shown in Figure 2.

**Figure 3:**
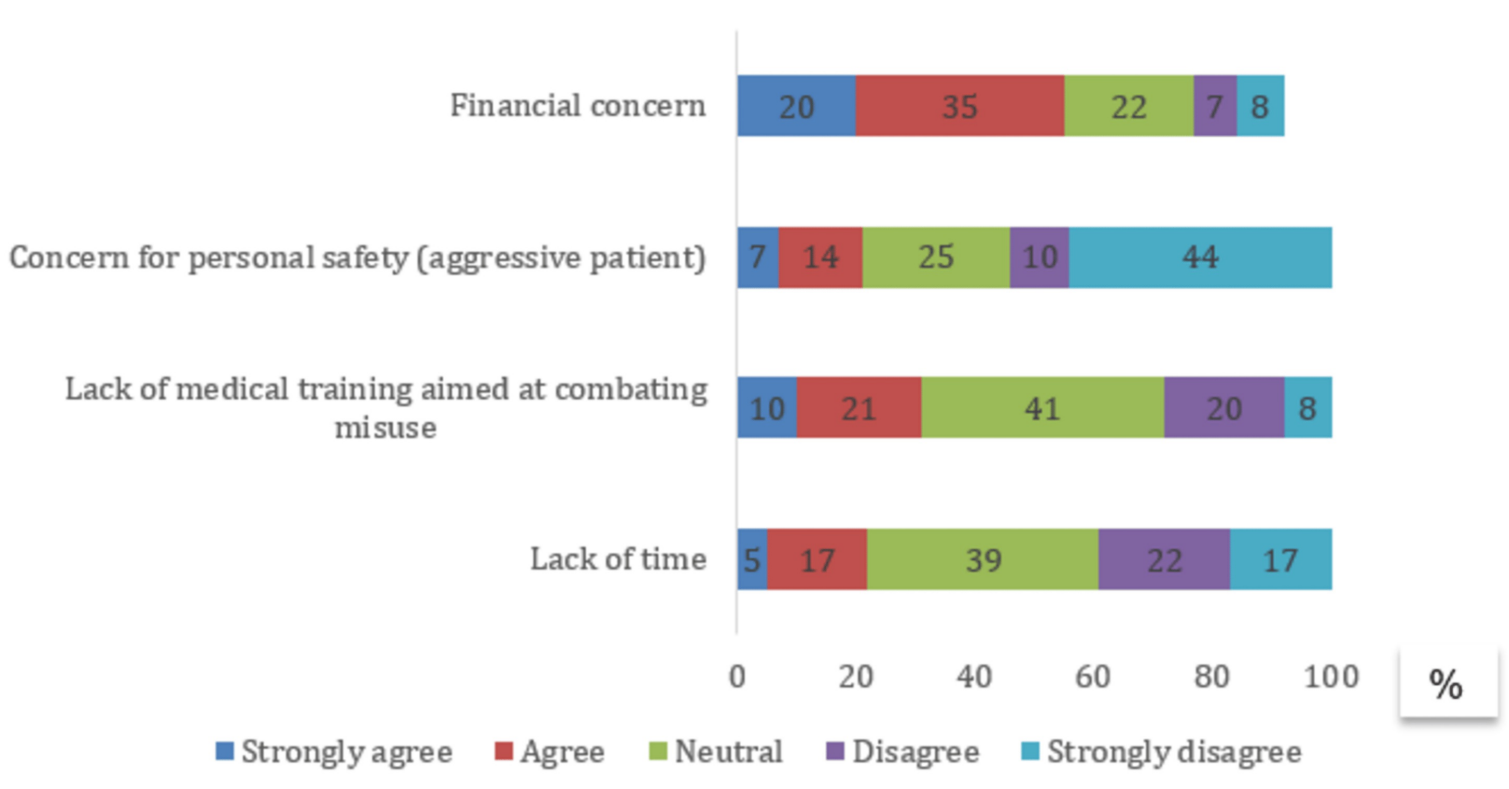
Challenges faced by community pharmacists in Morocco that hinder the fight against NSAID misuse (N = 100) NSAID: non-steroidal anti-inflammatory drug

About 55% agreed that financial problems, especially the risk that patients might buy their medicines elsewhere, are a major challenge to stopping NSAID misuse. Other challenges are shown in Figure 3.

To combat NSAID misuse, all pharmacists supported the implementation of specific legal restrictions, including stricter enforcement of prescription-only regulations, control of OTC sales, and sanctions for non-compliance. Furthermore, 97% of respondents favoured awareness campaigns to educate the public about the risks of misuse and to enhance patient care. A large majority (86%) agreed on the need for stronger social security coverage, such as improved reimbursement and access to safer therapeutic alternatives. Additionally, 81% supported increased reporting of adverse drug reactions (ADRs) to pharmacovigilance authorities, and 82% believed pharmacists should have easier access to patients’ medical records.

Practices

In daily practice, 64% of pharmacists dispensed NSAIDs without a prescription. This occurred in various situations: when patients experienced pain (70%), were regular customers (66%), had financial difficulties limiting access to care (44%), faced difficulty accessing healthcare (43%), refused to consult a doctor (23%), or displayed aggressive behaviour (6%).

Before dispensing NSAIDs without a prescription, most community pharmacists took precautionary measures to ensure patient safety. The majority of these pharmacists informed patients about the maximum treatment duration (85%) and the recommended dosage (84%). A significant proportion also checked for potential contraindications (70%) and warned patients about possible side effects (61%). Additionally, 59% verified the absence of drug interactions before supplying the medication.

If misuse was suspected, 64% of pharmacists warned patients about the risks. About half refused to sell NSAIDs (49%), nearly half offered safer OTC alternatives (44%), and 25% said the medication was not available. Only 6% of pharmacists reported NSAID-related adverse reactions to the CPAM.

Pharmacists in urban areas were more likely to inform patients about treatment duration and maximum dose (p = 0.03) and to check contraindications before dispensing NSAIDs without a prescription (p = 0.04), compared to those in peri-urban or rural areas. They also more often warned patients about misuse risks and offered safer OTC options (p = 0.02).

Some peri-urban and rural subgroups included a small number of participants (5 to 7), which should be taken into account when interpreting the statistical comparisons. Other factors like age, gender, or years of practice did not significantly affect pharmacists’ practices. Detailed results of pharmacists’ practices are shown in Table 2.

**Table 2:** Practices of community pharmacists in Morocco regarding NSAID dispensing. NSAID: non-steroidal anti-inflammatory drug; CPAM: National Pharmacovigilance Centre Number (percentage %). Statistical test used: Chi-square test for categorical variables

Practices	Workplace location			Total (N = 100)	Statistic value (χ²)	p-value
	Urbain	Peri-urban	Rural			
I dispense NSAIDs without a prescription.	52 (62.7)	5 (62.5)	7 (77.8)	64 (64)	0.81	0.66
I inform patients about the maximum NSAID dosage not to exceed.	71(85.5)	8 (100)	5(55.6)	84 (84)	7.09	0.03
I inform patients about the maximum duration for taking NSAIDs.	72 (86.7)	8 (100)	5 (55.6)	84 (84)	7.73	0.03
I check for contraindications before dispensing NSAIDs without a prescription.	58 (69.9)	8 (100)	4(44.4)	70 (70)	6.23	0.04
I inform patients about the potential side effects of NSAIDs.	51 (61.4)	7 (87.5)	3 (33.3)	61 (61)	5.26	0.07
Before dispensing NSAIDs, I check for any potentially risky drug interactions.	49 (59)	7 (87.5)	3 (3.33)	59 (59)	5.14	0.07
In case of NSAID misuse, I refuse to sell the medication.	42 (50.6)	5 (62.5)	2 (22.2)	49 (49)	3.25	0.19
In case of NSAID misuse, I deny the availability of the medication.	22 (26.5)	2 (25)	1 (1.11)	25(25)	1.03	0.59
In case of NSAID misuse, I educate the patient about proper dosage, duration, potential side effects, and interactions.	53 (63.9)	8 (100)	3 (33.3)	64 (64)	8.17	0.02
In case of NSAID misuse, I suggest appropriate and safe over-the-counter alternatives based on patient age, comorbidities, and potential drug interactions.	35 (42.2)	7 (87.5)	2 (22.2)	44 (44)	7.99	0.02
Patients come to the pharmacy to report side effects related to NSAID misuse.	11(13.3)	3(37.5)	1(11.1)	15 (15)	3.48	0.17
I report the side effects reported by my patients to the CAPM.	6 (7.2)	0 (0)	0(0)	6 (6)	1.31	0.52

Note: Percentages for precautionary measures refer only to pharmacists who dispensed NSAIDs without a prescription. 

## Discussion

This descriptive study provides valuable insight into how community pharmacists in Morocco understand and manage the misuse of NSAIDs. It reveals a gap between theoretical knowledge and practical experience, reflecting the complex role pharmacists play in balancing professional responsibilities, healthcare system limitations, and patient expectations.

The results show that most pharmacists have a good general understanding of NSAID misuse and its main adverse effects. Studies from other countries, such as Qatar, have similarly shown that pharmacists possess strong knowledge about the gastrointestinal risks of these drugs [12]. In Morocco, our findings indicate comparable awareness; however, knowledge gaps remain, particularly regarding the legal classification of NSAIDs (List I, List II, OTC). These gaps may influence dispensing practices, potentially leading to deviations from regulations and patient safety risks. Addressing them through targeted training and access to updated reference materials is therefore crucial, especially within the Moroccan pharmacy context.

In practice, NSAIDs are frequently dispensed without a prescription, often in response to acute pain, patient loyalty, or limited access to healthcare. While these behaviors reflect pharmacists’ attempts to meet patient needs, they also underscore potential safety concerns. Similar patterns have been observed in Thailand, where even non-OTC NSAIDs are often provided without prescription [13]. These observations suggest that systemic pressures and barriers to formal care contribute to self-medication practices, highlighting the importance of context-specific interventions.

Pharmacists commonly provide advice on dosage, treatment duration, and contraindications, aligning with established good practice standards [12]. However, fewer consistently counsel on side effects and drug interactions, which may limit the educational impact for patients. This indicates an area where targeted interventions could reinforce safe use practices.

When misuse is suspected, pharmacists adopt a range of strategies: explaining risks, refusing inappropriate requests, suggesting safer alternatives, or indicating that the medication is unavailable. Rather than simply reporting willingness to prevent misuse, these actions reveal how pharmacists translate knowledge into practice, and how gaps in classification knowledge can affect decision-making. Pharmacists with a better understanding are more likely to guide patients on dosage, treatment duration, and safe alternatives, demonstrating that knowledge directly influences both attitudes and behaviors.

The study also highlights differences based on workplace location. Good practices were more frequent in urban areas, likely reflecting better professional organization, access to training, and resource availability. These findings suggest that interventions should be region-specific, with targeted support for rural and peri-urban pharmacists to strengthen safe dispensing. Nevertheless, small sample sizes in some subgroups (5-7 participants) mean these results should be interpreted with caution, as statistical power is limited.

Another important finding is the low rate of ADR reporting to the CAPM. Despite the availability of simple reporting channels, this underreporting weakens pharmacovigilance. Comparative data from Thailand show higher ADR reporting for NSAIDs [14], highlighting opportunities to enhance monitoring and feedback systems in Morocco.

Pharmacists in our study identified the main reasons patients misuse NSAIDs: acute pain, lack of knowledge about overdoses, or misunderstanding proper use, which aligns with previous research linking low health literacy and easy access to NSAIDs to misuse [15]. The integration of knowledge, attitudes, and practices observed in our study shows that gaps in pharmacists’ knowledge, particularly regarding legal classifications, can directly affect their decisions and guidance, emphasizing the need for targeted training. This is especially important in Morocco, where chronic and inflammatory pain is prevalent, and patient education remains uneven [16].

Despite systemic challenges, pharmacists demonstrated proactive strategies to mitigate misuse, reflecting professional responsibility similar to findings in Egypt [17]. However, structural barriers, such as competition, financial pressure, risk of losing patients, and weak coordination with other healthcare professionals, limit the effectiveness of these efforts.

To address these challenges, pharmacists proposed practical solutions, including national awareness campaigns, enhanced social security support, and clearer legislation on NSAID dispensing. These recommendations represent professional insights rather than evidence from intervention studies, and future research should evaluate their effectiveness in reducing NSAID misuse in the Moroccan context.

This study has several strengths. It is the first descriptive study in Morocco to comprehensively examine pharmacists’ knowledge, attitudes, and practices regarding NSAID misuse. It provides a detailed understanding of current practices and highlights areas where interventions could improve public health.

Some limitations must be considered. The sample of 100 pharmacists may not fully reflect national practices. Self-reported data are subject to bias, including socially desirable responses or recall errors. Additionally, the cross-sectional design precludes causal inference or tracking changes over time. Nevertheless, the study provides original insights that can guide decisions on pharmacist training, regulatory updates, and support systems to ensure the safe use of NSAIDs in the general population.

## Conclusions

This study highlights several important points about NSAID misuse in community pharmacies. It shows that pharmacists have strong knowledge about misuse, but NSAIDs are often dispensed without a prescription, and adverse drug reaction reporting remains low. Pharmacists play a key role in preventing misuse, yet they face challenges related to limited training, regulatory knowledge, and patient counseling.

To address these issues, interventions should be prioritized and clearly guided. Targeted training programs on NSAID classification and safe dispensing practices should be implemented first, followed by structured awareness campaigns and support from social security to improve patient access to formal healthcare. Strengthening pharmaceutical counseling and establishing practical systems in pharmacies to monitor dispensing and follow-up could provide measurable outcomes, such as increased adherence to guidelines and reduced inappropriate NSAID use.

Finally, this study has limitations that should be considered when interpreting the results. The sample size of 100 pharmacists may not fully represent national practices, the data are self-reported and subject to bias, and the cross-sectional design does not allow assessment of causality or changes over time. Future research should aim to address these limitations and evaluate the effectiveness of proposed interventions in the Moroccan context.

## Appendices

Questionnaire

A - Data on the Pharmacist

1. Age (in years):

2. Sex: ☐ Male   ☐ Female

3. Years of experience as a pharmacist (since obtaining the Doctor of Pharmacy degree):

4. Years of practice in community pharmacy:

5. Location of practice as a community pharmacist: ☐ Urban area   ☐ Rural area   ☐ Suburban area

6. Socioeconomic level of the neighborhood: ☐ High   ☐ Middle   ☐ Low

B - Knowledge

1. Misuse is defined as the intentional and inappropriate use of a medication that is not in accordance with its marketing authorization (MA): ☐ True   ☐ Probable   ☐ Unlikely   ☐ False   ☐ I don’t know 2. Misuse refers to inappropriate use in terms of: - Indication: ☐ True   ☐ Probable   ☐ Unlikely   ☐ False   ☐ I don’t know - Dosage: ☐ True   ☐ Probable   ☐ Unlikely   ☐ False   ☐ I don’t know - Duration: ☐ True   ☐ Probable   ☐ Unlikely   ☐ False   ☐ I don’t know 3. Dispensing NSAIDs should be done cautiously in patients at cardiovascular risk. ☐ True   ☐ Probable   ☐ Unlikely   ☐ False   ☐ I don’t know 4. Dispensing NSAIDs should be done cautiously in patients at risk of peptic ulcer disease. ☐ True   ☐ Probable   ☐ Unlikely   ☐ False   ☐ I don’t know 5. In Morocco, pyrazoles, indoles, and oxicams are classified as List I drugs: ☐ True ☐ Probable ☐ Unlikely ☐ False ☐ I don’t know 6. In Morocco, arylcarboxylic acids and fenamates are classified as List 2 drugs. ☐ True   ☐ Probable   ☐ Unlikely   ☐ False   ☐ I don’t know 7. In Morocco, salicylates, ibuprofen, and ketoprofen are available over the counter (OTC). ☐ True   ☐ Probable   ☐ Unlikely   ☐ False   ☐ I don’t know

C - Attitudes

1.     I believe that pharmacists play an important role in the fight against drug misuse.

☐ Strongly agree ☐ Agree ☐ Neutral ☐ Disagree ☐ Strongly disagree

2. Obstacles and challenges that hinder the fight against NSAID misuse in Morocco: ☐ Financial concerns (patients can obtain the medication elsewhere) ☐ Personal safety concerns (aggressive patients) ☐ Lack of medical training aimed at combating misuse ☐ Lack of time 3. Measures considered necessary to reduce NSAID misuse in Morocco: ☐ Awareness campaigns on the risks of drug misuse ☐ Improving the patient care pathway ☐ Enhancing social security coverage ☐ Strengthening adverse drug reaction (ADR) reporting ☐ Facilitating pharmacists’ access to patients’ medical records ☐ Enforcing government regulations to reduce misuse

D - Practices

Please indicate how often you perform the following practices: 1. I dispense NSAIDs without a prescription.

☐ Always   ☐ Often   ☐ Sometimes   ☐ Rarely   ☐ Never

2. I dispense NSAIDs without a prescription in the following situations: ☐Patient in severe pain ☐Patient suffering (moderate pain) ☐Regular client of the pharmacy ☐Financial barriers to medical care ☐Difficulty accessing medical appointments ☐Patient refuses to see a physician ☐Aggressive patient 3. I advise patients to take the lowest effective NSAID dose.

☐ Always   ☐ Often   ☐ Sometimes   ☐ Rarely   ☐ Never

4. I inform patients about the recommended duration of NSAID use not to exceed.

☐ Always   ☐ Often   ☐ Sometimes   ☐ Rarely   ☐ Never

5. I check for contraindications before dispensing NSAIDs.

☐ Always   ☐ Often   ☐ Sometimes   ☐ Rarely   ☐ Never

6. I explain to patients the potential adverse effects related to NSAID use.

☐ Always   ☐ Often   ☐ Sometimes   ☐ Rarely   ☐ Never

7. I check for possible drug interactions, particularly concomitant NSAID use.

☐ Always   ☐ Often   ☐ Sometimes   ☐ Rarely   ☐ Never 8. When faced with a situation of misuse: ☐I refuse to sell the medication. ☐I deny the availability of the medication. ☐I educate the patient about the risks of misuse. ☐ I suggest safer over-the-counter alternatives. 9. I receive reports from patients about adverse effects related to NSAID misuse.

☐ Always   ☐ Often   ☐ Sometimes   ☐ Rarely   ☐ Never 10. I report adverse effects to the Moroccan Poison Control and Pharmacovigilance Center (CAPM).

☐ Always   ☐ Often   ☐ Sometimes   ☐ Rarely   ☐ Never
 
